# A Two-Step Protocol for Isolation and Maintenance of Lung Cancer Primary 3D Cultures

**DOI:** 10.3390/cancers17010027

**Published:** 2024-12-25

**Authors:** Silvia Strocchi, Giacomo Santandrea, Eleonora Zanetti, Giulio Verna, Vincenza Ylenia Cusenza, Davide Nicoli, Valentina Fantini, Alessandra Grieco, Massimiliano Paci, Alessia Ciarrocchi, Valentina Sancisi

**Affiliations:** 1 Translational Research Laboratory, Azienda Unità Sanitaria Locale-IRCCS di Reggio Emilia, 42122 Reggio Emilia, Italy; silvia.strocchi88@gmail.com (S.S.); giulio.verna@ausl.re.it (G.V.); valentina.fantini@ausl.re.it (V.F.); alessandra.grieco@ausl.re.it (A.G.); alessia.ciarrocchi@ausl.re.it (A.C.); 2 Pathology Unit, Azienda Unità Sanitaria Locale-IRCCS di Reggio Emilia, 42122 Reggio Emilia, Italy; giacomo.santandrea@ausl.re.it (G.S.); eleonora.zanetti@ausl.re.it (E.Z.); vincenzaylenia.cusenza@ausl.re.it (V.Y.C.); davide.nicoli@ausl.re.it (D.N.); 3 Biobank, Azienda Unità Sanitaria Locale-IRCCS di Reggio Emilia, 42122 Reggio Emilia, Italy; 4 Clinical and Experimental Medicine PhD Program, University of Modena and Reggio Emilia, 41100 Modena, Italy; 5 Thoracic Surgery Unit, Azienda Unità Sanitaria Locale-IRCCS di Reggio Emilia, 42122 Reggio Emilia, Italy; massimiliano.paci@ausl.re.it

**Keywords:** lung cancer, organoids, 3D cultures, protocol, CTOS, spheroids

## Abstract

Background/Objectives: Despite the introduction of innovative therapeutics, lung cancer is still the leading cause of cancer-related death. For this reason, lung cancer still requires deep characterization to identify cellular and molecular targets that can be used to develop novel therapeutic strategies. Three-dimensional cellular models, including patient-derived organoids (PDOs), represent useful tools to study lung cancer biology and may be employed in the future as predictive tools in therapeutic decisions. However, the successful establishment of lung cancer organoids cultures that faithfully represent the respective patient tissues is still challenging due to low success rate and/or overgrowth of normal airway epithelial cells. Methods: We set up a two-step protocol that allows for establishing both short-term and long-term 3D cultures, with different characteristics and success rates. Results: Cancer tissue-originated spheroids (CTOSs) show a 100% success rate and allow for the concomitant isolation of autologous tumor infiltrating leukocytes (TILs). On the contrary, PDOs can be expanded for a medium-long term and bio-banked but retain a lower success rate and the possibility of contamination with normal airway epithelial cells. To overcome these problems, we set up an optimal medium formulation and we implemented rigorous quality controls, leading to a substantial improvement in the success rate of tumoral PDO establishment. Conclusions: Overall, this protocol guarantees flexibility and reliability, also providing useful guidelines for quality control checks to support different experimental settings. The setting up of a robust protocol for lung cancer PDO culture establishment and expansion is a key requirement for their employment both in cancer research and as predictive tools in clinical practice.

## Simple Summary

Patient-derived organoids are 3D models useful to study cancer biology and may be implemented as predictive tools in therapeutic decisions. We set up a two-step protocol that allows for establishing both short-term and long-term organoid cultures from lung cancer patients. We set up an optimal medium formulation and implemented rigorous quality controls, leading to a substantial improvement in organoid establishment success rate. Our 3D models are suitable for drug screening and co-culture experiments. This protocol represents a key advance for the employment of lung cancer organoids both as models for research and as predictive tools in clinical practice.

## 1. Introduction

Despite significant advancements in lung cancer treatment, this disease remains the main contributor to tumor mortality [1]. In the last years, there have been notable improvements in therapeutic options, but the choice of optimal treatment is still restrained by the tumor heterogeneity, the insurgence of drug resistance, and the lack of reliable biomarkers, leading to limitations in patient outcomes [2].

In this context, the employment of patient-derived three-dimensional (3D) models, including spheroids and organoids, can be useful in studying the biology of tumors in a patient-specific manner, maintaining 3D architecture, heterogeneity, and, in some circumstances, also interactions with the microenvironment [3]. Furthermore, 3D models offer a platform to assess drug sensitivity, providing a predictive tool that may be implemented in therapeutic decisions. In line with this, patient-derived 3D models from different cancer types, including colon, breast, pancreas, and kidney, have been demonstrated to be relatively manageable and useful tools both to study tumor biology and to predict drug sensitivity [4,5,6,7,8,9]. Indeed, several clinical trials are currently ongoing, aiming to assess the feasibility of organoids employment in a personalized medicine setting (e.g., NCT05183425; NCT03500068; NCT03577808).

However, the establishment of lung cancer organoids cultures is still challenging due to low success rates and/or overgrowth of normal airway organoids. Success rates have exhibited significant variability, ranging from 7% to 88%, depending on the study. In addition, reported instances of normal organoid overgrowth have pointed out the need for protocol optimization and reliable quality controls. Different groups have explored multiple growth media and/or strategies for tumoral organoid selection, including nutlin treatment and hand-picking, with variable results, leading to a lack of consensus on the optimal strategy to develop pure lung cancer organoid cultures [10,11,12,13,14,15,16].

In this work, we report our experience on the development of a two-step protocol that allows for the establishment of both short-term and long-term cultures, with different characteristics and different success rates. Cultures of cancer tissue-originated spheroids (CTOSs) show limited growth and can be maintained for a short period of time, but in turn hold a success rate of virtually 100%, preventing normal airway spheroid overgrowth and enabling the concomitant isolation of tumor infiltrating leukocytes (TILs). On the other hand, patient-derived organoids (PDOs) can be passaged for a medium–long period of time, expanded, and bio-banked, allowing for low- and medium-throughput drug screening, but still holding important drawbacks, including a low success rate and the need for rigorous quality controls. This two-step protocol guarantees flexibility, holding the possibility to perform short-term or long-term cultures, to support different experimental settings, and provides useful guidelines for quality control checks to ensure fidelity and purity of lung cancer organoid cultures. These aspects are fundamental to develop organoid-based research protocols and predictive tools.

## 2. Materials and Methods

### 2.1. Patients Enrollment

A total of 33 patients with new lung adenocarcinoma diagnosis at the Azienda USL-IRCCS of Reggio Emilia (Italy) from September 2021 to August 2023 were included in this study. Supplementary Table S1 summarizes patient clinicopathological features.

### 2.2. Isolation of CTOSs from Fresh Biopsies

Lung cancer surgical samples were carefully separated from contaminating stroma and weighted. After cutting in small pieces with a scalpel, the samples were incubated with 5 mL/g of tissue of digestion solution (50 µg/mL Liberase, 13.6 Kunitz U DNAse, 100 µg/mL penicillin/streptomycin in DMEM/F12) for at least 30 min at 37 °C in a shaker. To stop the enzymatic digestion, an equal volume of DMEM/F12 containing 20% FBS was added. The digested sample was centrifuged and resuspended in DMEM/F12. First, the suspension was filtered through a 100 µm strainer to remove large undigested fragments. Then, the suspension was filtered through a 40 µm strainer to separate the single cells (filtered) from the CTOSs that remained in the strainer. The strainer was inverted to retrieve the CTOSs by washing with DMEM/F12. CTOSs were centrifuged and resuspended in CTOS medium (100 µg/mL penicillin/streptomycin, 50 ng/mL EGF, 20 ng/mL bFGF, 1x B27, 6 U/mL sodium heparin) in case of use for co-cultures or in DMEM/F12 in case of further processing to obtain PDOs. CTOSs were transferred into a 96-well plate to set up co-cultures with TILs or into a 6-well plate and incubated for at least 4 h before further processing. For the full protocol and for detailed data on reagents, see Supplementary Information and Supplementary Table S2.

### 2.3. Concomitant Isolation of TILs from Fresh Biopsies and Co-Cultures with CTOSs

Single cells filtered through a 40 µm strainer can be used to isolate TILs. Depending on the presence of red blood cells, the flow-through was centrifuged and resuspended in red blood cells lysis solution (0.8% NH_4_Cl) for 5 min at 4 °C. The suspension was centrifuged again and resuspended in 4–5 mL of DMEM. TILs were separated from other single cells using a Ficoll gradient (Histopaque-1077, MERCK, Darmstadt, Germany). TILs were resuspended in TIL medium (10% FBS, 100 µg/mL penicillin/streptomycin, 1 µg/mL interleukin-2 in RPMI). TILs were counted and added to CTOS cultures to start co-culturing. Before starting the co-cultures, TILs can be stained with Cytolight Red for imaging purposes. For the full protocol and for detailed data on reagents, see Supplementary Information and Supplementary Table S2.

### 2.4. Flow Cytometry Analysis of TILs

Part of the isolated TILs were further characterized by flow cytometry. Briefly, 5 × 10^5^ cells were pelleted and resuspended in cold staining buffer (PBS 1X, BSA 0.5%). Antibody mix was then added to the cell suspension and incubated for 30 min at 4 °C in the dark. 7-AAD staining was used to gate live cells. TILs were acquired using the BD FACS Canto II instrument (BD, Franklin Lakes, NJ, USA) and BD FACSDIVA v8 software and further analyzed using Kaluza v2.1. For the full list of antibodies and complete protocol, see Supplementary Information and Supplementary Table S2.

### 2.5. CTOS Processing to Start PDO Cultures

After at least 4 h of culture in suspension, CTOSs were processed to isolate single cells and to start PDO cultures. CTOSs were centrifuged and resuspended in 1–3 mL of TrypLE, then incubated at 37 °C for 10–15 min in a shaker. To stop the digestion, an equal volume of DMEM/F12 with 20% FBS was added. Samples were centrifuged and resuspended in DMEM/F12. Cells were counted, centrifuged again, and resuspended in organoid culture medium. Basement Membrane Extract (BME, BME001-10, Biotechne, Minneapolis, MN, USA) was added at the final concentration of 70%. The volume was calculated to generate 50 µL domes containing 1.5–2 × 10^4^ cells each. Each 50 µL drop was seeded in a well of a 24-well plate (3524, Corning, Corning, NY, USA) and kept 1 min right side up at room temperature and 9 min upside-down at 37 °C to create the domes. A quantity of 600 µL organoid culture medium was added to each well and the plate was incubated in the cell incubator. For the full protocol and for detailed data on reagents, see Supplementary Information and Supplementary Table S2.

### 2.6. Organoids Splitting

Organoids cultures were split when organoids reached an average diameter of 100–200 μm. The organoid culture medium was removed from wells and each dome was resuspended in 2 mL TrypLE. Samples were incubated at 37 °C for 15–25 min in a shaker. To stop the digestion, an equal volume of DMEM/F12 with 20% FBS was added. Samples were washed once with DMEM/F12 and resuspended in the correct volume of organoid culture medium to split the culture. Depending on the growing rate of the cultures, they can be split at a 1:2–1:4 ratio. BME at a final concentration of 70% was added to the organoid suspension and 50 μL domes were generated as previously described.

### 2.7. Organoids Freezing and Thawing

Organoids were frozen when they reached an average diameter of 20–30 µm. The organoid culture medium was removed from the well and the dome was mechanically disrupted by pipetting up and down in cold DMEM/F12. Samples were centrifuged, resuspended in Recovery Cell Culture freezing medium and transferred in cryovials. Cryovials were slowly frozen using an isopropanol freezing container and then moved in the liquid nitrogen tank. The organoid suspension was thawed in a pre-warmed bath. A quantity of 1–2 mL of DMEM/F12 was added and the suspension was centrifuged. The pellet was washed with 5–6 mL of DMEM/F12, centrifuged again, and then resuspended in organoid culture medium. BME was added at the final concentration of 70%, as previously described.

### 2.8. Organoids Paraffin Inclusion and Staining

PDO cultures that have reached an average diameter of 100 µm can be processed for paraffin inclusion and staining. The culture medium was removed, and the domes were washed with cold PBS 1X; 1 mL of 4% paraformaldehyde (PFA) was added directly into the well to dissolve the matrix. The suspension was moved to a 1.5 mL tube and incubated in a shaker at room temperature overnight. The day after, the sample was centrifuged and washed once with cold PBS 1X. The pellet was resuspended in 50 µL of 2% agarose. A drop was generated and let solidify. Then, it was embedded in paraffin in a biopsy embedding cassette. The sample was cut in 4 µm slides and stained with hematoxylin/eosin or specific immunohistochemistry (IHC) antibodies. Morphology and IHC profiles of PDO samples were compared with original tumors. For the full protocol and for detailed data on reagents, see Supplementary Information and Supplementary Table S2.

### 2.9. TP53 Sequencing

The DNA obtained from FFPE patient’s sample was used for TP53 analysis by Sanger sequencing; 1 μL of DNA sample was amplified by polymerase chain reaction (PCR) with TaqGo polymerase (Roche CustomBiotech, Mannheim, Germany) and previously published primer pairs [17].

The validation was performed by 2% agarose gel electrophoresis. The PCR products were purified by GeneJET PCR Purification Kit (ThermoFischer Scientific, Waltham, MA, USA). Subsequently, the sequencing was executed using BigDye Terminator v3.1 (Roche CustomBiotech, Mannheim, Germany). Following the purification carried out by PERFORMA^®^ Gel Filtration Cartridge (EdgeBio, San Jose, CA, USA), an electrophoretic run on an 8-capillary ABI Prism^®^ 3500DX Genetic Analyser (ThermoFischer Scientific, Waltham, MA, USA) was carried out. Sequencing results were analyzed using the “Sequencher” software program version 5.4.6 (Genecodes, Ann Arbor, MI, USA). For detailed protocol and primer sequences, see Supplementary Information.

### 2.10. Mutational Analysis

During the first or second organoid split, 100 μL of the cell suspension after TrypLE digestion was transferred in a 1.5 mL tube and centrifuged. The pellet was washed with 500 μL PBS 1X and then the DNA was extracted using the DNeasy Blood and Tissue kit (QIAGEN, Germantown, MD, USA), following the manufacturer’s protocol. DNA samples were quantified and sequenced using the Myriapod NGS Cancer panel DNA (Diatech, Jesi, Italy). Mutational profiles of PDOs were compared with tumor mutational profiles of original patients obtained for diagnostic purposes, using the same panel.

### 2.11. Organoid Growth Curves and Drug Screening

After validation of the PDO culture, one confluent dome was digested with TrypLE to obtain organoids of small dimensions (20–30 µm diameter). As already described, the digestion was stopped, and the pellet was washed once with DMEM/F12. To generate PDO growth curves with or without the addition of drugs, 2000 small organoids were added for each well of a 96-well plate, on the top of a thin layer of BME at 50% concentration previously dried at 37 °C for 30 min. The organoid growth was monitored, placing the plate into the Incucyte S3 Live Imaging instrument (Sartorius, Gottingen, Germany). Images were taken every 24 h and analyzed using the organoids module (Sartorius, Gottingen, Germany). Three days after seeding, drugs were added to the organoid cultures. The changes in organoid area were normalized to time zero acquisition and standard errors were calculated for each group of replicates.

### 2.12. Statistical Analysis

Statistical analyses were performed using GraphPad Prism Software version 8.0 (GraphPad Software, Boston, MA, USA). Statistical significance was determined using two-tailed Student’s *t* test. Adjusted *p* value was calculated by correcting the *p* value for multiple testing using Benjamini–Hochberg’s method.

## 3. Results

### 3.1. Establishment of CTOS as Short-Term Primary 3D Cultures

**Figure 1:**
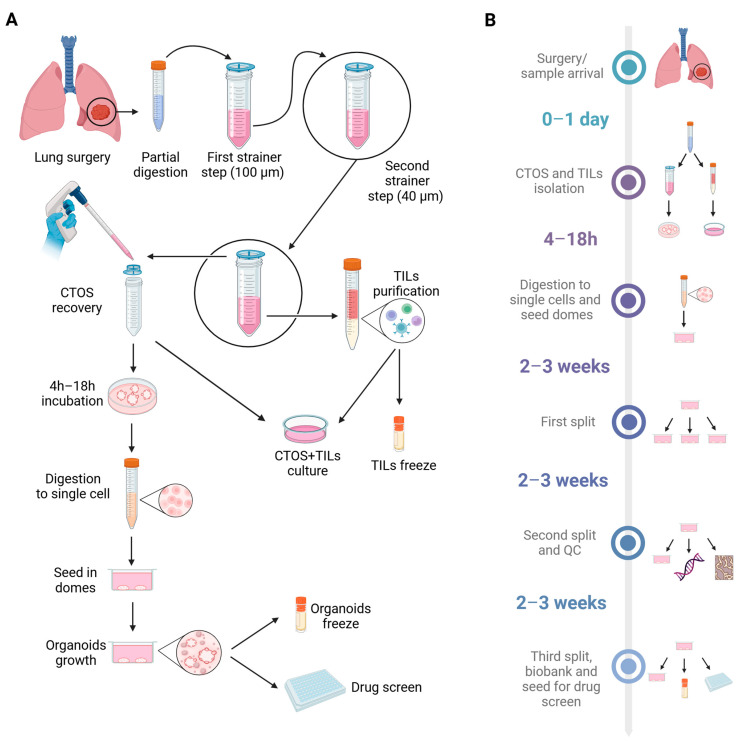
Scheme of the experimental protocol. (**A**) Overview of the experimental protocol. Lung cancer tissue is subjected to a partial digestion. After a first filtering step through a 100 μm strainer, the sample is filtered through a 40 μm strainer. At this stage the filtered single cell suspension can be further purified through Ficoll sedimentation to isolate TILs. The partially undigested tissue (CTOSs) can be retrieved from the strainer and used for co-cultures with TILs. Alternatively, CTOS culture is further processed after 4 h to a single-cell suspension and seeded in BME domes to generate an organoid culture. (**B**) Timeline of the experimental procedure of PDO culture, from sample collection to bio-banking and drug screen. QC: quality checks. Created in BioRender.com.

**Figure 2:**
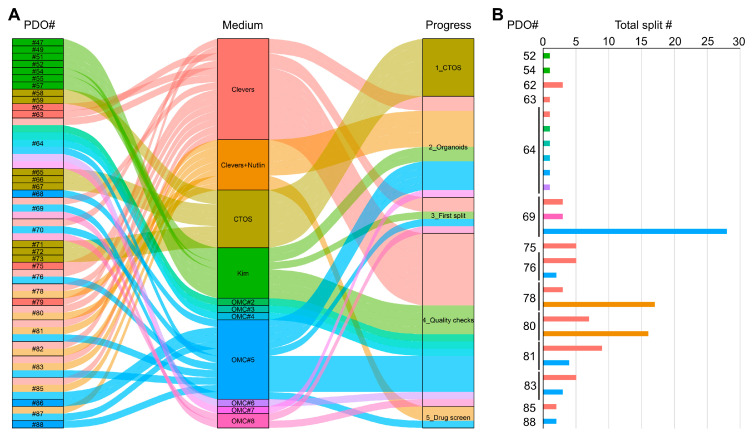
Overview of culture progress in our primary cultures collection. For each of the 33 samples used to setup this protocol, the culture medium and the progress in culture phases is indicated (**A**). Culture media are indicated for each PDO and represented in different colors. In (**B**), lifespan, indicated in split number, is reported for each PDO. Bar colors correspond to culture media as in panel A.

From September 2021 to August 2023, 33 samples deriving from surgical resection of lung adenocarcinomas were collected (Figure 1 and Figure 2). Considering the low success rate and the issues with normal airway organoid overgrowth that have been reported in the literature [14,15], we decided to take advantage of a short-term 3D culture protocol that we previously employed [18] to generate CTOSs. CTOSs are derived from partially digested cancer tissue, retaining original cell–cell interaction, and not requiring a growth phase in matrix to be used. Notably, these cultures are not subjected to cell growth pressure; therefore, they maintain the heterogeneity typical of a tumor. The protocol includes two filtering steps through strainers with different diameters, also allowing for the retrieval of single cells from the tissue, which can be further processed to isolate TILs. Furthermore, the maintenance of the CTOS culture in suspension for at least 4 h allows for the attachment of fibroblasts and other contaminating cell types to the bottom of the plate and the subsequent removal by transferring CTOSs to a new vessel. CTOS cultures can be successively digested to a single-cell suspension and included in a 3D matrix, such as BME or Matrigel, starting a proper PDO culture (Figure 1).

**Figure 3:**
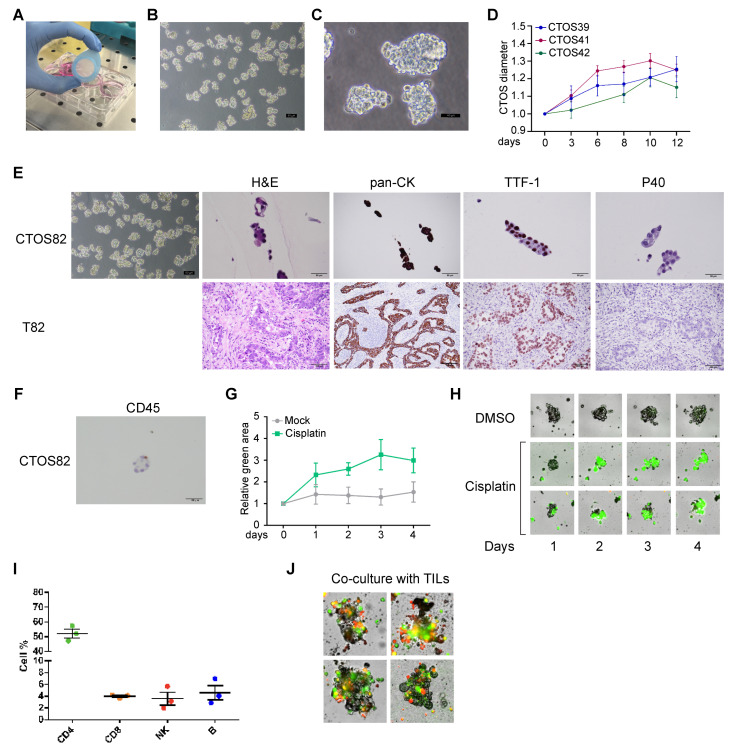
Establishment of CTOS cultures from lung cancer surgical samples. (**A**) Picture of the key strainer filtering step. (**B**,**C**) Pictures of representative CTOS cultures at two different magnifications (100× and 400×). (**D**) Growth curves of three representative CTOS samples. (**E**) Quality check analysis of CTOS82. Phase contrast microscope picture—hematoxylin/eosin (H&E), pancytokeratin (panCK), TTF-1, and p40 staining of CTOS82 and corresponding patient sample T82 are shown. (**F**) CD45 staining of CTOS82, showing an immune cell infiltrating the CTOSs. (**G**) Analysis of cisplatin-induced cytotoxicity in CTOS culture. Green fluorescent area measuring Annexin V signal was normalized on time 0 for each sample. (**H**) Pictures of CTOSs treated with 100 µM cisplatin or mock-treated and stained with Annexin V green fluorescent staining. (**I**) Flow cytometry analysis of surface markers in three TIL samples. (**J**) Pictures of CTOSs in co-cultures with TILs. Green fluorescence indicates Annexin V staining while red fluorescence marks the TILs.

Using this method, we successfully established CTOS cultures from 33 out of 33 processed samples, obtaining a 100% success rate (Figure 2). These cultures have limited growth potential and tend to degenerate within 2–3 weeks (Figure 3A–D). However, due to the scarce proliferation, the overgrowth of minority subpopulations or normal airway cells is also unlikely. To confirm this notion, we verified the concordance of CTOSs with the original patients through a quality check, including (1) hematoxylin/eosin (H&E) staining followed by histological morphology analysis; (2) IHC on specific markers; (3) NGS analysis of 16 cancer-relevant genes. For IHC analysis, we used pancytokeratin (pan-CK), the most sensible and specific marker of generic epithelial lineage; TTF-1, typically positive in lung adenocarcinoma (but not in squamous cell carcinoma); and p40, which stains both squamous cell carcinoma (but not adenocarcinoma) samples and healthy bronchial cells (see Supplementary Figure S1A for staining controls). As shown in Figure 3E, the analyzed CTOS cultures showed concordance with the original patient, regarding histological features and specific markers, while the mutational analysis resulted negative for all genes both in the patient’s original tissue and in the CTOSs. In addition, we confirmed the presence of immune-derived cells inside CTOSs by staining with CD45 (Figure 3F), indicating that CTOSs may also contain tumor microenvironment-derived cells. Collectively, our quality checks confirmed that CTOS cultures closely resemble the original tissue, indicating their potential use for personalized medicine. The quantity of CTOSs obtained was in the range of 200–500 per patient, depending on the amount of the starting material, being largely insufficient for medium- or high-throughput pharmacological screenings. However, we were able to confirm sensitivity to cisplatin (Figure 3G,H) and other drugs. In addition, this protocol allows for the isolation of TILs from the same patient. We obtained TILs in the range of 1 × 10^5^–3 × 10^6^/100 mg of processed tissue that showed a typical distribution of surface markers (Figure 3I and Figure S2A). These TILs allowed for the establishment of autologous co-cultures for TIL functionality analysis experiments (Figure 3J), as already reported in a previous publication [18].

### 3.2. Defining the Optimal Medium for Long-Term Organoids Cultures

Starting from 25 of 33 CTOS culture samples, we proceeded to the establishment of PDOs. The CTOSs were digested to a single-cell suspension and included in a BME matrix. Initially, we used the growth medium described by Kim and colleagues [11], assuming that a medium with fewer growth factors would favor the growth of cancer organoids over normal airway cells. Although the organoids cultured in this medium exhibited the solid morphology typical of the tumor type, their growth was very limited, and they were easily contaminated with fibroblasts (see Supplementary Figure S2B–D). Due to these problems, we switched to a medium with more growth factors to better stimulate the growth of cancer-initiating cells. The formulation developed by Hans Clevers’ laboratory is the most widely used and seems to warrant the best success rate, even if the overgrowth of normal airway organoids is a strong drawback that limits the use in cancer research [10]. We decided to proceed with this medium, maintaining stringent quality controls to check the concordance of PDO cultures with patient tumors. As already reported for CTOS analysis, these quality checks included H&E staining, IHC analysis of specific markers, and NGS analysis of a panel of cancer-related genes. All samples were analyzed and compared with original tumor with the help of a trained pathologist.

**Figure 4:**
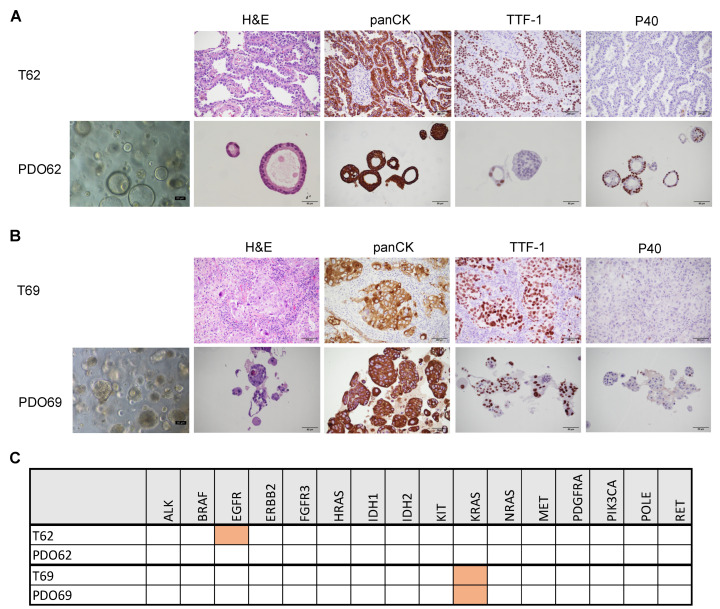
Representative lung PDO quality checks. (**A**) H&E, panCK, TTF-1, and p40 staining of tumor sample T62 or corresponding organoid culture PDO62. For PDO62, the phase contrast image is also shown. (**B**) H&E, panCK, TTF-1, and p40 staining of tumor sample T69 or corresponding organoid culture PDO69. For PDO69, the phase contrast image is also shown. (**C**) Table showing the mutational analysis of a panel of cancer-related genes in T62, PDO62, T69, and PDO69. Orange color indicates the presence of oncogenic mutation.

Figure 4 shows two examples of a PDO culture that passed (PDO69) and a PDO culture that did not pass (PDO62) the quality checks. PDO62 showed predominant cystic morphology upon H&E staining, resembling a normal lung tissue composed by ciliated cells surrounding a lumen often filled with mucus (Figure 4A). On the contrary, PDO69 showed a solid morphology and other features, such as enlarged nuclei or hyper-pigmented cells, typical of tumoral samples (Figure 4B). For IHC analysis, we used the same panel of markers used in the previous section for CTOS analysis. Both PDO69 and PDO62 derived from surgical resections for adenocarcinoma and thus are expected to be positive for pan-CK and TTF-1 and negative for p40. As shown in Figure 4B, PDO69 shows concordance of all three markers between the PDO and the corresponding patient cancer tissue, being strongly positive for pan-CK, positive for TTF-1, and negative for p40. On the contrary, PDO62 showed strong staining for pan-CK, but negativity for TTF-1 and positivity for p40 (Figure 4A). This would indicate that the PDO62 organoids are derived either from a minor squamous component of the tumor or from normal airway cells. Notably, by definition the squamous component should be less than 10% of tumor to qualify the lesion as adenocarcinoma with a minor squamous component and not adenosquamous carcinoma. As further quality control check, we performed NGS analysis on a set of 16 cancer-related genes on both original patient tumor samples and PDO samples. The T69 patient showed a high frequency of KRAS G12C mutation, which was maintained in PDO69, whereas T62 carried an EGFR mutation that was retained in PDO62 at a very low frequency, indicating a massive contamination with non-tumoral cells (Figure 4C).

After the quality check analyses on PDO62, which was cultured in Clevers medium, we decided to test various media formulations (Organoid Media Combination OMC#2–8), combining changes in different factors in order to eliminate, or at least limit the normal airway organoids outgrowth. Two samples, PDO64 and PDO69, were subdivided and cultured in parallel in different media, named OMC#2–8, representing variants of the two media previously used [10,11]. The composition of OMC#2–8 media is shown in Supplementary Table S3, in comparison with Clevers and other published lung cancer organoid media [10,11,12,13,16].

**Figure 5:**
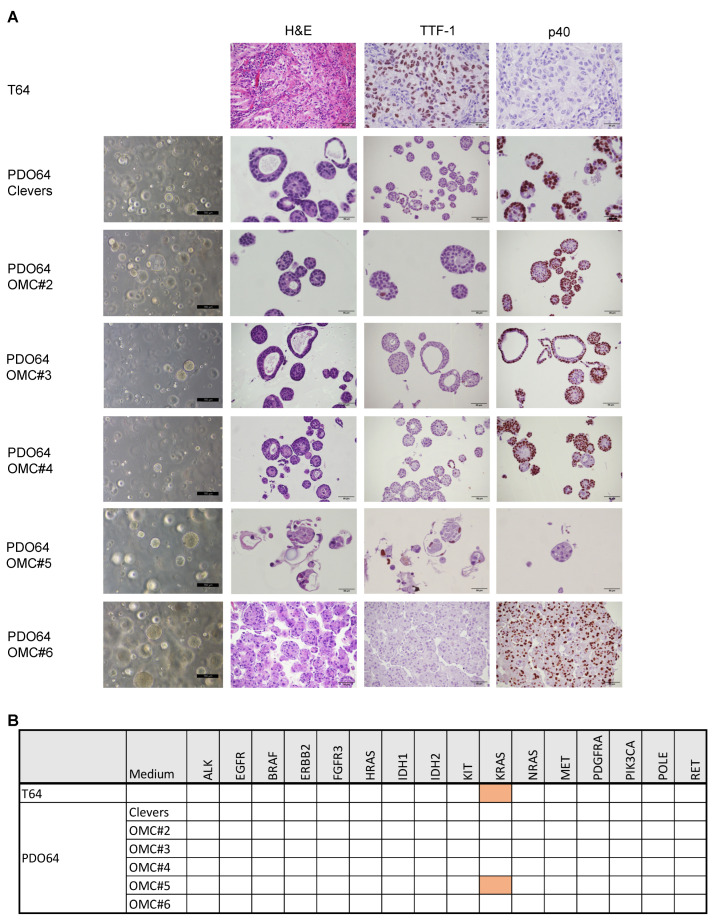
Quality checks of PDO64 cultured in different organoid media combinations. (**A**) H&E, TTF-1, and p40 staining of tumor sample T64 or corresponding organoid culture PDO64. For PDO64, phase contrast images are also shown. PDO64 was cultured in the indicated media variants (OMC#2–6). (**B**) Table showing the mutational analysis of a panel of cancer-related genes in T64 and PDO64 cultured in the different media variants. Orange color indicates the presence of oncogenic mutation.

**Figure 6:**
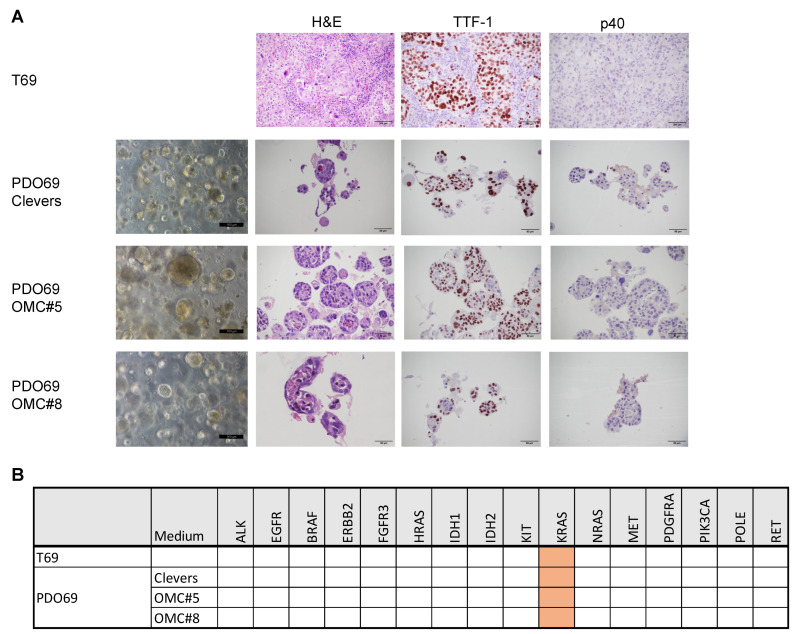
Quality checks of PDO69 cultured in different organoid media combinations. (**A**) H&E, TTF-1, and p40 staining of tumor sample T69 or corresponding organoid culture PDO69. For PDO69, phase contrast images are also shown. PDO69 was cultured in the indicated media variants (OMC#5,8). (**B**) Table showing the mutational analysis of a panel of cancer-related genes in T69 and PDO69 cultured in the different media variants. Orange color indicates the presence of oncogenic mutation.

Notably, all the tested media produced an organoid culture, except PDO64 in OMC#7, but showing different characteristics, regarding, for example, the dimensions and the number of the 3D structures and the presence of fibroblasts or adherent cell contamination (Figure 5A). All samples displaying growing organoids after the first split were subjected to quality checks to assess concordance with the original tumor (Figure 2 and Supplementary Table S4). In case of PDO64, only organoids grown in medium OMC#5 showed full concordance with the patient sample (Figure 5A,B). Regarding PDO69, concordance with the patient was shown in three different growing media, including Clevers and OMC#5 (Figure 6A,B). These results may suggest that OMC#5 is more selective for tumoral organoids, the optimal formulation also being dependent on patient-specific factors. For these reasons, we decided to compare OMC#5 with Clevers medium and to grow the subsequent organoid cultures in parallel with different medium formulations: Clevers, OMC#5, and Clevers + nutlin-3a. Nutlin-3a is toxic to cells that are wild-type for TP53; thus, the addition of this drug is used to counter-select normal airway organoids [10,14]. Indeed, we verified nutlin-3a selectivity in a panel of NSCLC cell lines, confirming that this drug restrains the growth only in the TP53 wild-type context (Supplementary Figure S2E,F). This strategy is particularly helpful in the lung cancer context, considering that the percentage of patients carrying a TP53 mutation is around 50% [19].

### 3.3. Establishment of PDO for Long-Term Primary 3D Cultures

Based on the results obtained with different media combinations, the following samples were cultured in Clevers, OMC#5, Clevers + nutlin-3a, or in parallel with more than one of these media. Excluding the samples that were used as CTOSs and those grown in Kim medium, a total of 18 PDOs were cultured with one or more organoid medium combinations, as summarized in Table 1, Table 2 and Table S4. In 11 cases, we obtained a proliferative culture with at least one of these media (success rate of growth 61%). These cultures underwent IHC and NGS quality checks to establish correspondence with patient tissue. Fourteen PDOs were cultured in Clevers medium; five did not proliferate; the others underwent the quality checks but only two passed them (success rate of growth 71%; success rate of quality check pass 20%). Eleven PDOs were cultured in OMC#5; five did not proliferate enough to undergo the quality checks, whereas five out of the six PDOs that showed active growth passed both IHC and NGS quality checks (success rate of growth 55%; success rate of quality check pass 84%). Seven PDOs were grown in Clevers + nutlin-3a; five cases showed an extremely low proliferative rate that impeded the performance of the quality checks; two cases passed the controls, showing correspondence with patient genetic background and tumor phenotype (success rate of growth 29%; success rate of quality check pass 100%) (Table 2).

These numbers, as summarized in Table 1, Table 2 and Table S4, indicate that PDOs grown in Clevers medium show a high success rate of establishment (71%) but a low quality check pass rate (20%). Notably, nutlin-3a treatment inhibited the growth of more than half the samples, in line with a TP53 mutation percentage of about 50% in lung adenocarcinoma. TP53 sequencing confirmed the mutational status of the PDOs that survived nutlin-3a treatment (Table 1 and Table S4). Strikingly, PDOs grown in OMC#5 showed a lower growth rate (55%), compared to those grown in Clevers medium, but a higher quality check pass rate (84%) (Table 2). These data indicate that OMC#5 is more selective than Clevers medium for lung tumor organoids. Overall, 18 PDOs were cultured in one or more organoid media combinations, showing growth in at least one condition in 11 cases (61%) and passing quality checks in at least one condition in 7 cases (38%) (Table 2).

**Figure 7:**
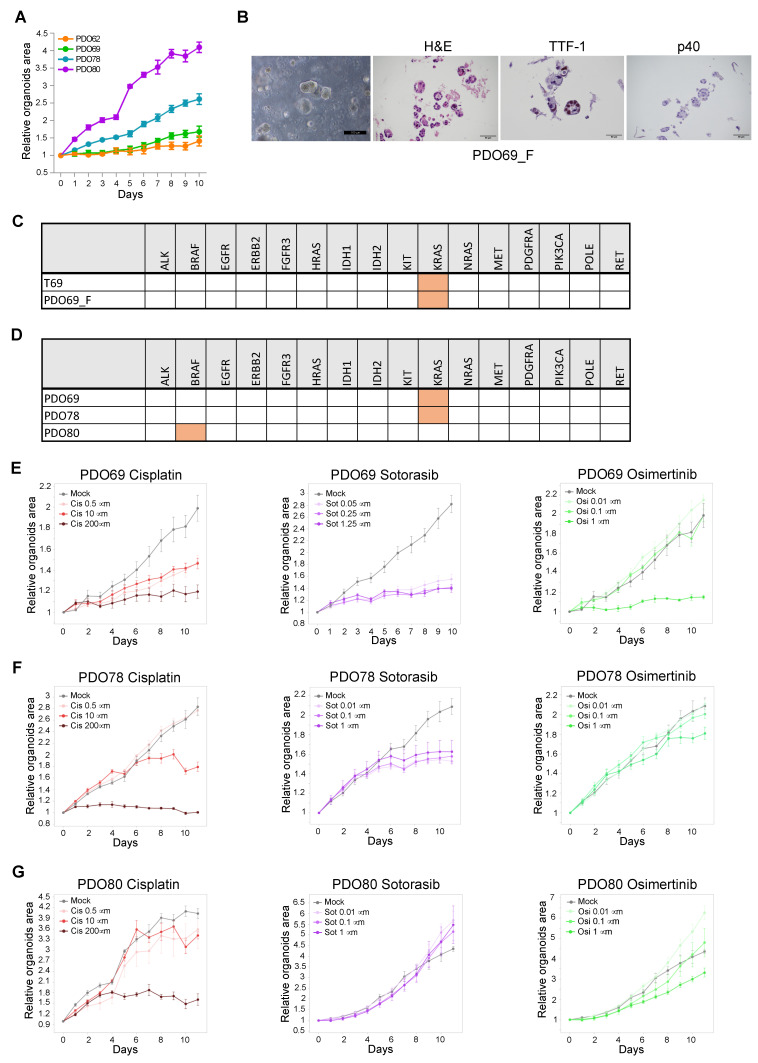
Long-term culture of lung PDO and drug sensitivity screen. (**A**) Growth curves of PDO62, PDO69, PDO78, and PDO80 (n = 5; technical replicates). (**B**) Phase contrast image and H&E, TTF-1, and p40 staining of PDO69 after 13 split and one cycle of freeze/thaw (PDO69_F). (**C**) Table showing the mutational analysis of a panel of cancer-related genes in T69 and PDO69_F. Orange color indicates the presence of oncogenic mutation. (**D**) Mutational analysis of PDO69, PDO78, and PDO80. Orange color indicates the presence of oncogenic mutation. (**E**–**G**) Drug sensitivity curves of PDO69, PDO78, and PDO80 treated with increasing concentrations of cisplatin, osimertinib, or sotorasib. Total organoid area was measured at each time-point and normalized on the area at time 0. Values are expressed as mean ± SEM (standard error of the mean) (n = 5; technical replicates).

Notably, both normal airway PDOs and lung cancer PDOs showed active growth and a number of splitting passages between 1 and more than 20 (Figure 2 and Figure 7A and Table 1). Eight samples were bio-banked, and the success rate of recovery from liquid nitrogen was verified for six of them. One sample, PDO69, was still growing after more than 18 months in culture and was re-tested for concordance with original tumor after a freeze/thaw cycle at split #13. Remarkably, it still showed high concordance of both histological features and mutational profile (Figure 7B,C). Next, PDO69, PDO78, and PDO80 were tested for sensitivity to a panel of lung cancer-specific anti-cancer drugs (cisplatin, sotorasib, and osimertinib). In accordance with their genetic profile (Figure 7D), PDO69 and PDO78, showing KRAS G12C mutation, displayed high sensitivity to KRAS inhibitor sotorasib and low sensitivity to EGFR inhibitor osimertinib (Figure 7E,F). PDO80, being wild-type for both KRAS and EGFR, showed resistance to both specific inhibitors (Figure 7G). The three PDOs showed variable sensitivity to cisplatin (Figure 7E–G). These results suggest that PDOs generated with our protocol are suitable for drug screening analysis.

## 4. Discussion

The results described in this study present a comprehensive approach to the establishment and validation of CTOSs as short-term primary 3D cultures and PDOs as long-term primary 3D cultures for studying lung adenocarcinoma. These findings align with and contribute to the existing literature on the development of 3D culture models for cancer research. In the context of lung cancer, there is still a lack of consensus on the best conditions to isolate and propagate organoid cultures, leading to a limited application of this technology. In the present work, we report the establishment of short-term cultures (CTOSs) with a noteworthy success rate of 100%. The use of a short-term 3D culture protocol minimizes the risk of outgrowth of minority subpopulations or normal airway cells, overcoming the issues presented by multiple authors [12,14,15]. The ability of CTOS cultures to maintain the original cell–cell interactions in 3D cultures is another important point supporting the use of this model for studying the complexity of tumors. In addition, the inclusion of a method for isolating TILs from the same patient sample and establishing autologous co-cultures for TIL functionality analysis is a novel and valuable aspect of this methodology, as it allows for studying the tumor microenvironment. On the other hand, the yield of CTOS isolation and the time they can be kept in culture are major drawbacks of this procedure, limiting the application for medium–high-throughput experiments.

Thus, the transition from CTOS to long-term PDO cultures is a critical step to allow for culture expansion and bio-banking. Our findings indicate that the choice of culture medium plays a pivotal role in the success of PDO establishment. Indeed, Clevers medium, though widely used, can result in the overgrowth of normal airway organoids. On the other hand, using a medium containing fewer growth factors, like the one suggested by Kim et al. [11], may promote the generation of tumor organoids but in a time window that leads to fibroblast overgrowth. Notably, we observed that while organoids derived from some patients showed different characteristics in different media, organoids derived from other patients grew efficiently and with good fidelity to the original tumor in all the tested media (compare, e.g., PDO64 with PDO69 in Figure 5 and Figure 6). These findings suggest the existence of still uncharacterized patient-specific factors that influence the success rate of the culture. Although, in our hands, OMC#5 is the most selective medium combination for lung tumor organoids, these patient-specific factors make it difficult to define a growing medium that is optimal for all patients. To overcome this problem, whenever it was possible, we cultured the samples in different media in parallel and selected the best conditions for each patient, based on stringent quality checks. This approach allowed for us to increase success rate of PDO growth, compared with using OMC#5 alone, and simultaneously to increase the percentage of samples passing the quality checks, compared with using Clevers medium alone.

Our data also highlight the need for stringent quality controls to be performed on all samples, including histological, immunohistochemical, and genetic characterization, to ensure that PDO cultures accurately represent the patient’s tumor. Indeed, the enormous discrepancy in the success rate of lung cancer organoid culture establishment reported in the literature, ranging from 7% to 88% [12,15], may be due to different culture conditions but also to different methods of considering a successful culture. For example, the study by Shi et al. reported an 88% rate of organoid culture establishment, but also that 75% of primary organoids were contaminated with normal airway cells, lowering the success rate of uncontaminated cancer cultures to 22% [12]. The study by Dijkstra et al. reports a success rate for culture establishment of 41% that lowers to 17% when only considering organoid samples that passed the quality checks [14]. In this context, we defined a successful organoid culture as one that gave enough material to perform the quality checks (usually performed after 2–3 split) and the quality check pass as the correspondence with the original tumor in both IHC and mutational analysis. Given these definitions, our success rates for organoid culture establishment of 61% and for quality check pass of 38% represent an improvement compared to the literature (Table 2).

Both CTOSs and PDOs were shown to be suitable for drug screening, although the limited growth of CTOSs makes them suitable for low-throughput experiments only. The high sensitivity of PDO69 and PDO78 to KRAS inhibitor sotorasib and their low sensitivity to EGFR inhibitor osimertinib is in line with their genetic profile, while PDO80, being wild-type for both KRAS and EGFR, showed resistance to both inhibitors. These results indicate that using PDO cultures for testing the efficacy of lung cancer-specific drugs is feasible and suggest that PDOs offer a valuable tool for tailoring treatment strategies to individual patients based on their tumor’s molecular characteristics.

In this context, the limited success rate and the contamination with normal airway cells remain important drawbacks for the clinical application of organoids as a predictive tool. To overcome these issues, other groups reported the use of alternative sources for patient tumor material, such as lymph node metastases and malignant pleural effusion [20,21]. Although these sources may not be available for all patients, the thorough comparison of organoids derived from them with those derived from primary tumor tissues may be a future avenue of investigation.

## 5. Conclusions

Overall, the results of this study contribute to the growing body of knowledge in the field of 3D culture techniques. The combination of short-term CTOS cultures and long-term PDO cultures provides a comprehensive approach for studying lung adenocarcinomas and other tumors and represents a significant step forward in cancer research. In addition, our results highlight the need for rigorous quality control to establish organoid cultures representing original tumors with a high confidence level. The implementation of a reliable protocol for organoid establishment and expansion is a critical step for their wide utilization as tools both for cancer research and for drug response prediction in clinical settings.

## Figures and Tables

**Table 1:** Summary of organoid growth in media variants.

Patient #	Histology	TP53	Medium	Growth	Split #	Quality Checks	
						IHC	NGS
62	ADK	n/a	Clevers	Y	3	X	X
63	ADK	n/a	Clevers	N	1	n/a	V
64	ADK	n/a	Clevers	Y	1	X	X
			OMC#2	Y	1	X	X
			OMC#3	Y	1	X	X
			OMC#4	Y	1	X	X
			OMC#5	Y	1	V	V
			OMC#6	Y	1	X	X
			OMC#7	N	1	n/a	n/a
68	ADK	n/a	OMC#5	N	2	n/a	n/a
69	ADK	n/a	Clevers	Y	3	V	V
			OMC#5	Y	21	V	V
			OMC#8	Y	3	V	V
70	ADK	n/a	Clevers	N	0	n/a	n/a
			OMC#5	N	0	n/a	n/a
			OMC#8	N	0	n/a	n/a
75	ADK	n/a	Clevers	Y	5	X	X
76	ADK	n/a	Clevers	Y	5	X	X
			OMC#5	Y	2	X	X
78	ADK	MUT	Clevers	Y	3	X	X
			Clevers + nutlin-3a	Y	9	V	V
79	ADK	WT	Clevers	N	0	n/a	n/a
80	ADK	MUT	Clevers	Y	7	V	V
			Clevers + nutlin-3a	Y	7	V	V
81	ADK	WT	Clevers	Y	10	X	V
			Clevers + nutlin-3a	N	1	n/a	n/a
			OMC#5	Y	4	V	V
82	ADK	WT	Clevers	N	1	n/a	n/a
			Clevers + nutlin-3a	N	1	n/a	n/a
83	ADK	WT	Clevers	Y	7	X	V
			Clevers + nutlin-3a	N	0	n/a	n/a
			OMC#5	Y	3	V	V
85	ADK	WT	Clevers	Y	3	X	X
			Clevers + nutlin-3a	N	0	n/a	n/a
			OMC#5	N	1	n/a	n/a
86	ADK	WT	OMC#5	N	1	n/a	n/a
87	ADK	MUT	OMC#5	N	1	n/a	n/a
			Clevers + nutlin-3a	N	1	n/a	n/a
88	ADK	WT	OMC#5	Y	2	V	V

ADK: adenocarcinoma; WT: wild-type; MUT: mutated; Clevers: Clevers laboratory growing medium as reported in Sachs et al. [10]; OMC#2–8: organoid media combinations, as indicated in Supplementary Table S3; Y: yes; N: no; X: quality check not passed; V: quality check passed; n/a: not available.

**Table 2:** Success rate of different organoid culture conditions.

Medium	Total PDO	Growth + PDO	% Growth + PDO	QC Pass PDO	% QC on Total PDO	% QC on Growth + PDO
Clevers	14	10	71%	2	14%	20%
Clevers + nutlin-3a	7	2	29%	2	29%	100%
OMC#5	11	6	55%	5	45%	84%
Any medium	18	11	61%	7	38%	63%

Clevers: Clevers laboratory growing medium as reported in Sachs et al. [10]; OMC#5: organoid medium combination #5, as indicated in Supplementary Table S3; Any medium: number of organoids showing growth or passing quality checks in at least one of the considered conditions; PDO: patient-derived organoid; QC: quality check.

## Data Availability

All data supporting the findings of this study are available within the paper and its Supplementary Information.

## Supplementary Materials

The following supporting information can be downloaded at: https://www.mdpi.com/article/10.3390/cancers17010027/s1, Supplementary Information: Supplementary Materials and Methods; Table S1: Clinical and histopathological features of lung cancer patients; Table S2: List of reagents; Table S3: Summary of media composition; Table S4: Quality checks, Figure S1: IHC staining controls; Figure S2: Additional controls.
