# Supplementary material for: A Two-Step Protocol for Isolation and Maintenance of Lung Cancer Primary 3D Cultures
Source: Cancers (Basel). 2024 Dec 25;17(1):27. doi: 10.3390/cancers17010027 (PMC11718983; doi:10.3390/cancers17010027)
Supplement: Supplementary file 1 [file cancers-17-00027-s001.zip › Strocchi et al Supplementary Information.docx]

**A two-step protocol for isolation and maintenance of lung cancer primary 3D cultures**

Silvia Strocchi, Giacomo Santandrea, Eleonora Zanetti, Giulio Verna, Vincenza Ylenia Cusenza, Davide Nicoli, Valentina Fantini, Alessandra Grieco, Massimiliano Paci, Alessia Ciarrocchi, Valentina Sancisi

**Supplementary Materials and Methods**

**Detailed protocol for organoids handling**

***Reagents and solutions***

*Note: All the reagents and resources are listed in Supplementary* ***Data 2****.*

[Final concentration]

DIGESTION SOLUTION

- Advanced DMEM/F12 medium
- [50 μg/ml] Liberase
- [13.6 U/ml] DNase
- [100 μg/ml] Penicillin/Streptomycin

Filter the solution with a 0.22 mm filter. Store at -20°C up to 3 months.

SHORT TERM CULTURING SOLUTION (CTOS medium):

- DMEM/F12 medium
- [100μg/mL] Penicillin/Streptomycin
- [50ng/mL] human EGF
- [20ng/mL] bFGF
- [1x] B27
- [6 U/mL] Sodium heparin

Store at 4°C up to 2 weeks.

LONG TERM CULTURING SOLUTION (Clevers medium):

- Advanced DMEM/F12 medium
- [5 μM] Y-27632
- [500 nM] A83-01
- [500 nM] SB202190
- [1x] B27
- [50x] N2 supplement
- [50 ng/ml] human EGF
- [1.25 mM] N-Acetylcysteine
- [5 mM] Nicotinamide
- [0.5x] Glutamax supplement
- [10 mM] Hepes Buffer
- [100 U/ml] Antibiotic/Antimycotic

Store at 4°C up to 2 weeks.

BASEMENT MEMBRANE EXTRACT (BME)

Place the new tube of BME in a 4°C refrigerator for at least 10 h. Invert the tube several times to mix well the BME. Put 1.5 microcentrifuge tubes on ice to cool down. Distribute 500 μl of BME in each microcentrifuge tubes on ice and store the aliquots at -80°C for 6 months.

*Note: P1000 tips should be pre-wetted and pre-cooled by pipetting up and down cold sterile PBS + 0.1% BSA for several times before aspirating BME to prevent the matrix from sticking to the tips.*

TILs GROWING MEDIUM

- RPMI medium
- [100 μg/ml] Penicillin/Streptomycin
- [10%] Fetal Bovine Serum (FBS)
- [1 μg/ml] IL-2

The following paragraphs will describe the sequential steps necessary to set up the 3D cultures. We distinguish a short-term culture, composed by Cancer Tissue Originated Spheroids (CTOS), and a long-term culture made by organoids. All supports used are 24 well plates, different supports are specified.

***Establishment of lung adenocarcinoma 3D cultures from biopsies***

*Note: before starting it is necessary to place BME on ice to gently thaw the matrix; prewarm the culture media at room temperature; thaw the digestion solution.*

1. Weight the sample before starting to decide the correct volume of digestion solution to be used. 5 ml of digestion solution are necessary for each gram of tissue.
2. Put the sample on a 10 cm diameter Petri dish. Using a scalpel cut the tissue in small pieces till it is obtained a fine paste.
3. Using a sterile Pasteur put the sample with the correct amount of digestion solution in a 15ml conical tube and incubate at 37°C shaking for at least 30 min.
4. Each 10 min vortex the solution.

*Note: the digestion should stop when the suspension becomes turbid. DO NOT exceed the 80 min.*

1. To stop the enzymatic digestion, add an equal volume of DMEM/F12 containing 20% FBS.
2. Centrifuge at 650 g for 5 min.
3. Wet the 100 μm strainer with DMEM/F12.
4. Remove the supernatant and using a sterile Pasteur resuspend the pellet in 5-7 ml of DMEM/F12.
5. Smash the suspension through a 100 μm diameter strainer to eliminate the massive particles that are still present after the enzymatic digestion. Eliminate the filter.
6. Wet the 40 μm strainer with DMEM/F12.
7. Using a 40 μm diameter strainer, filter the eluted suspension to separate the single cells from the CTOS suspension.

*Note: the CTOS remain stuck in the filter pores, therefore KEEP the filter.*

*Note: to isolate the immunity components, KEEP the eluted fraction.*

1. Invert the strainer in a well of a six well and remove the CTOS from the filter pores, using DMEM/F12 + Penicillin/Streptomycin and a sterile Pasteur.

Depending on the type of analysis and on the quantity of material, at this point it is necessary to decide for a short-term culture, or a long-term culture. In the first case, CTOS are collected and seeded in the condition needed in CTOS medium (see the Co-cultures with autologous TILs paragraph). For the organoids culture see the Organoids cultures establishment paragraph.

***TILs isolation and biobanking***

1. Collect the flow-through (40 μm strainer) and centrifuge 450 g for 5 min (Single-cell suspension).
2. (Optional) To remove red blood cells add 1-3 ml of lysis solution (0.8% NH_4_Cl) and incubate for 5 min at 4°C.
3. Centrifuge 450 g for 5 min.
4. (Optional) If the pellet is still red, it is recommended to repeat red blood cell lysis with a shorter time of incubation of 2-3 min.
5. Resuspend the pellet in 4-5ml of DMEM/F12.
6. Set up Ficoll separation gradient.
7. Put 4 ml Ficoll in the bottom of a 15ml conical tube (Histopaque®-1077 sterile-filtered, density: 1.077 g/mL).
8. Using a sterile Pasteur add drop-by-drop the cell suspension from step 5.
9. Centrifuge 700 g for 30 min w/o brake.
10. After the centrifuge, collect the ring of cells in the interphase between the Ficoll and the solution, corresponding to TILs, through a sterile Pasteur.
11. Wash cells with PBS 1X and centrifuge 450 g for 10 min.
12. Re-suspend TILs in the TILs Growing Medium.
13. TILs may be analyzed fresh within 2 days or frozen in FBS+10% DMSO and stored in liquid nitrogen for subsequent analyses or for biobanking purposes.
14. Thaw in TILs Growing Medium and leave 2 days to recover before use.

***Flow cytometry analysis of TILs***

1. Gently thaw frozen TILs in a water bath, if starting from freshly isolated TILs skip this step.
2. Wash cells in PBS 1X + 2 mM EDTA + 1% BSA.
3. Count TILs and proceed to stain 5x10^5^ cells.
4. Pellet cells at 600 g for 5 min at 4°C.
5. Prepare the antibody mix in 50 μl of cold staining buffer (PBS 1X + BSA 0.5%).
6. Dispense the mix in each sample and incubate them at 4°C for at least 30 minutes in the dark.
7. Wash the TILs with 1 ml of staining buffer.
8. Pellet cells at 600 g for 5 min at 4°C.
9. Resuspend the pellet in 1 ml of PBS 1X and proceed to the instrument for the acquisition.

***Co-cultures of CTOS with autologous TILs.***

*Note: the following steps refer to a co-culture set up in 96well plate.*

1. Count viable TILs with Burker chamber.
2. Add 10^4^ TIL for each condition. Collect them and resuspend in 1 ml PBS 1X.
3. Add Cytolight Red at the final concentration of 0.5 μM. Keep the TIL in dark for 20 min at 37°C.
4. Add 500-600 μl of DMEM/F12+10% FBS to inhibit the staining solution.
5. Centrifuge at 600 g and resuspend in the correct volume of CTOS medium+ 1μg/ml IL-2.
6. Seed 30-100 CTOS in each well and add TILs when required by the experimental setting.

*Note: CTOS co-culture can be also tested for studying drug responsiveness. See paragraph below.*

***Organoids cultures establishment***

1. Leave the CTOS in DMEM/F12 in the 6 well for at least 4 hours at 37 °C in a CO_2_ incubator to permit the anchorage of the fibroblast and the single cell eventually present in the suspension.
2. At least 4 hours of incubation after isolation, collect the CTOS and centrifuge them at 450 g for 5 min.

*Note: leave the cells that adhere to the bottom in order to avoid contamination from fibroblasts.*

1. Remove the supernatant and resuspend the CTOS in 1-3 ml of TrypLE to create a single cell suspension.
2. Incubate the suspension at 37°C shaking for 10-15 min.
3. To stop the enzymatic digestion, add an equal volume of DMEM/F12 containing 20% FBS.
4. Centrifuge at 450 g for 5 min.
5. Remove the supernatant and resuspend the pellet in DMEM/F12 and count the cells using a Burker chamber. The domes should contain 1.5-2x10^4^ cells each.
6. Prepare the correct amount of BME and keep it on ice.
7. Centrifuge at 450 g for 5 min and resuspend the pellet in the correct volume of organoid medium.
8. Add the BME at the final concentration of 70% and pipette up and down carefully avoiding the creation of bubbles.
9. Seed 50 μl drop of the suspension in each well of a 24well plate. Keep it 1 min right side up at room temperature and 9 min upside-down at 37°C to create the domes.
10. Add 600 μl of organoid medium each well and let them growth at 37°C in a CO_2_ incubator.

*Note: the media should be changed twice a week leaving 100* μl *in the well and adding 500* μl *fresh.*

***Organoids splitting***

Once the organoids have reached an average dimension of 100-200 μm, it is necessary to split them in order to avoid fusions of the organoids and cell death at the center of the organoids.

1. Remove the media from the well and disrupt the matrix using 2ml of TrypLE.
2. Incubate at 37°C shaking for 15-25 min depending on the dimension of the organoids.
3. Add an equal volume of DMEM/F12 +20% FBS to stop the enzymatic reaction.
4. Centrifuge at 450 g for 5 min.
5. Remove the media and resuspend the pellet with 6-8 ml of DMEM/F12. Pipet up and down for 10-20 times.
6. Centrifuge at 450 g for 5 min.
7. Remove the supernatant and resuspend the pellet in the correct volume of organoid media to split the culture.

*Note: a low-density culture should be split 1:2, whereas a high-density culture should be split 1:4.*

1. Add the BME at the final concentration of 70% and pipette up and down carefully avoiding the creation of bubbles.
2. Seed 50 μl drop of the suspension in each well of a 24 well plate. Keep it 1 min right side up at room temperature and 9 min upside-down at 37°C to recreate the domes.
3. Add 600 μl of organoid media each well and let them growth at 37°C.

In order to verify that the organoids are representative of the original tumor, we set up two quality check controls (Histology and immunohistochemistry and Genetic analysis) to be performed before the experimentation starts and preferably within the second passage of the organoid culture.

***Histology and immunohistochemistry (IHC) of the organoid cultures***

For histology and IHC analysis use one dome from a 24 well plate:

*Note: the organoids should have reached an average dimension of 100 μm.*

1. Remove the media from the well and wash the dome with cold PBS 1X.
2. Add 1 ml of paraformaldehyde 4% (PFA) directly into the well to dissolve the matrix dome.
3. Pipet the suspension into a 1.5 ml tube and incubate shacking at room temperature over-night.
4. Centrifuge 6000 g for 5 min.
5. Remove the supernatant and wash the pellet with cold PBS 1X.
6. Prepare a 2% Agarose solution (dissolved in water)
7. Centrifuge 6000 g for 5 min.
8. Resuspend the pellet in 50 μl of Agarose solution using a 200 μl cut tip.
9. Let it solidify under chemical hood and then put it into a Biopsy embedding cassette.

The organoids are then stained with hematoxylin/eosin (H&E) or with the following antibodies with the IHC Roche Diagnostics procedure:

- P40 clone BC28 Mouse monoclonal antibody [0.4 μg/ml]
- TTF1 clone 8G7G3/1 Mouse monoclonal antibody [7 μg/ml]
- Anti Pan Keratin AE1/AE3/PCK26 Mouse monoclonal antibody cocktail [46.3 μg/ml]

***TP53 sequencing***

The DNA obtained from FFPE samples was used for the TP53 analysis by Sanger sequencing.

1. Amplification of 1μl DNA samples by Polymerase Chain Reaction (PCR) with TaqGo polymerase (Roche), following the amplification condition reported below.

PCR receipt for each sample:

- 1μl DNA Template
- 1μl primers 20μM (Forward+Reverse)
- 5μl Buffer5X
- 2.5μl MgCl2
- 1μl dNTP
- 0.25μl GoTaq
- 14.25μl H_2_O

PCR conditions:

| *Step* | *Cycles* | *Temperature* | *Time* |
| --- | --- | --- | --- |
| GoTaq Hot Start Polymerase activation | 1 | 95°C | 5 minutes |
| Denaturation | 35 | 95°C | 30 seconds |
| Annealing and extension |  | 62°C | 30 seconds |
| Elongation |  | 72°C | 30 seconds |
| Hold | 1 | 72°C | 5 minutes |
| Hold | 1 | 4°C | ∞ |

Primers:

| Name | Sequence |
| --- | --- |
| TP53 ex2-3 Forward | GGAGTGCTTGGGTTGTGGT |
| TP53 ex2-3 Reverse | CGGCAAGGGGGACTGTA |
| TP53 ex4 Forward | GACTTCCTGAAAACAACG |
| TP53 ex4 Reverse | CACACATTAAGTGGGTAAAC |
| TP53 ex5-6 Forward | TTTCTTTGCTGCCGTCTTC |
| TP53 ex5-6 Reverse | TTGCACATCTCATGGGGTTA |
| TP53 ex7 Forward | GACCATCCTGGCTAACGG |
| TP53 ex7 Reverse | CACAGGTTAAGAGGTCCCAAA |
| TP53 ex8-9 Forward | TTTGGGACCTCTTAACCTGT |
| TP53 ex8-9 Reverse | CAGGCAAAGTCATAGAACCAT |
| TP53 ex10 Forward | CATGTTGCTTTTGTACCGTC |
| TP53 ex10 Reverse | GGCAAGAATGTGGTTATAGGA |
| TP53 ex11 Forward | AAGGGAAGATTACGAGACT |
| TP53 ex11 Reverse | TAAGCTGGTATGTCCTACTC |

1. Validation by 2% agarose gel electrophoresis;
2. Purification of PCR products by GeneJET PCR Purification Kit (ThermoScientific) following the manufacturer’s protocol;
3. Sequencing executed using BigDye Terminator v3.1 (Roche);
4. Purification carried out by PERFORMA® Gel Filtration Cartridge (EdgeBio);
5. Electrophoretic run on 8-capillary ABI Prism® 3500DX Genetic Analyser (Applied Biosystems).

Sequencing results were analysed using the “Sequencher” software (Genecodes).

***Genetic analysis of organoids through NGS***

1. During the early organoid split, put 100 μl of the cell suspension after TrypLE digestion and put it in a 1.5ml tube.
2. Centrifuge 9400 g for 5 min.
3. Resuspend the pellet in 500 μl of PBS 1X.
4. Centrifuge 9400 g for 5 min.
5. Extract the DNA following the protocol of the DNeasy Blood and Tissue kit (Qiagen).
6. Quantify the sample with Nanodrop.
7. Follow the protocol of the Myriapod NGS Cancer panel DNA kit (Diatech)
8. Quantification of DNA input through Real-Time PCR
9. Amplification of the DNA fragments of interest
10. Clean-up: selection of PCR products
11. Add of specific index and adaptors Illumina
12. Normalization and preparation of the pool
13. Quantification through Qubit
14. Run the sequencing
15. Analyze the data

**Genes in the panel**

| **ALK** | **BRAF** | **EGFR** | **ERBB2** |
| --- | --- | --- | --- |
| **FGFR3** | **HRAS** | **IDH1** | **IDH2** |
| **KIT** | **KRAS** | **NRAS** | **MET** |
| **PDGFRA** | **PIK3CA** | **POLE** | **RET** |

***Organoids biobanking***

*Note: the organoids should be frozen 3 days after their split or when they reach an average diameter of 20-30 μm.*

1. Remove the media.
2. Collect the dome using cold DMEM/F12.
3. Pipette up and down few times to disrupt the matrix.
4. Centrifuge 450 g for 5 min.
5. Remove the media and resuspend the pellet in 1ml of Recovery Cell Culture freezing medium.
6. Leave it 5 min on ice and then put it at -80°C.
7. The day after move the vial into the nitrogen tank.

***Drug screening and cytotoxicity assay using 3D cell-cultures***

For CTOS co-culture:

Following the previously described method of CTOS isolating and co-culturing with TIL, it is also possible to test them for drug sensitivity.

1. Once the co-culture is seeded, add the drug in the desired concentration directly in the media of the co-culture.
2. Observe the culture for 3-7 days taking at least one photo per day.
3. Measure the dimensions of the CTOS manually and/or measure cytotoxicity using an enzymatic kit.

For organoids:

Note: prepare a 96 well plate coated with 40 μl of 50% BME in DMEM. The coated plate should dry at 37°C for 30 min.

1. Disrupt a confluent dome and incubate with TrypLE for 15 min to reduce the dimension of the organoids (around 20-30 μm is acceptable).
2. Count and seed 1500-2000 organoids per well (96 well plate).
3. Once the organoids have reached 40-60 μm dimensions (usually 2-5 days), drugs (single or combination) can be added to the supernatant.

*Note: change the media twice a week, paying attention to do not touch the bottom of the well and eventually scratch the BME layer.*

1. Organoids growth can be followed through different methods, including viability measurement through commercial kits. We used live cell imaging through Incucyte S3 instrumentation (Sartorius). Images have been collected at least once a day for 2 weeks and analyzed through the organoids software module (Sartorius). The analyses have been conducted creating a mask that takes into consideration both organoids size and eccentricity. For evaluating organoids growth, we considered the organoids area parameter.

***Cell cultures and Nutlin-3a treatments***

A549, NCI-H1650, NCI-H1975 and NCI-H2030 cell lines were grown at 37°C/5% CO_2_ in RPMI with 10% FBS and antibiotics. All cell lines were authenticated by SNP profiling at Multiplexion GmbH. Cells were tested for mycoplasm infection with a monthly schedule.

For Nutlin-3a treatment, 3000 A549 cells, 4000 NCI-H1650, 4000 NCI-H1975 and 2500 NCI-H2030 cells were seeded in quadruplicate in a 96-well culture plate. Nutlin-3a was added at 10 µM final concentration, and the cell proliferation was measured with Incucyte S3 live cell imaging system (Sartorius) for 72 hours. DMSO was used as Nutlin-3a treatment control.

**Supplementary Tables**

**Supplementary Table 1.** **Clinical and histo-pathological features of lung cancer patients**

Supplementary Table 1.xlsx

**Supplementary Table 2.** **List of reagents**

Supplementary Table 2.xlsx

**Supplementary Table 3.** **Summary of media composition**

Supplementary Table 3.xlsx

**Supplementary Table 4.** **Quality checks**

Supplementary Table 4.xlsx

**Supplementary Figures**

**
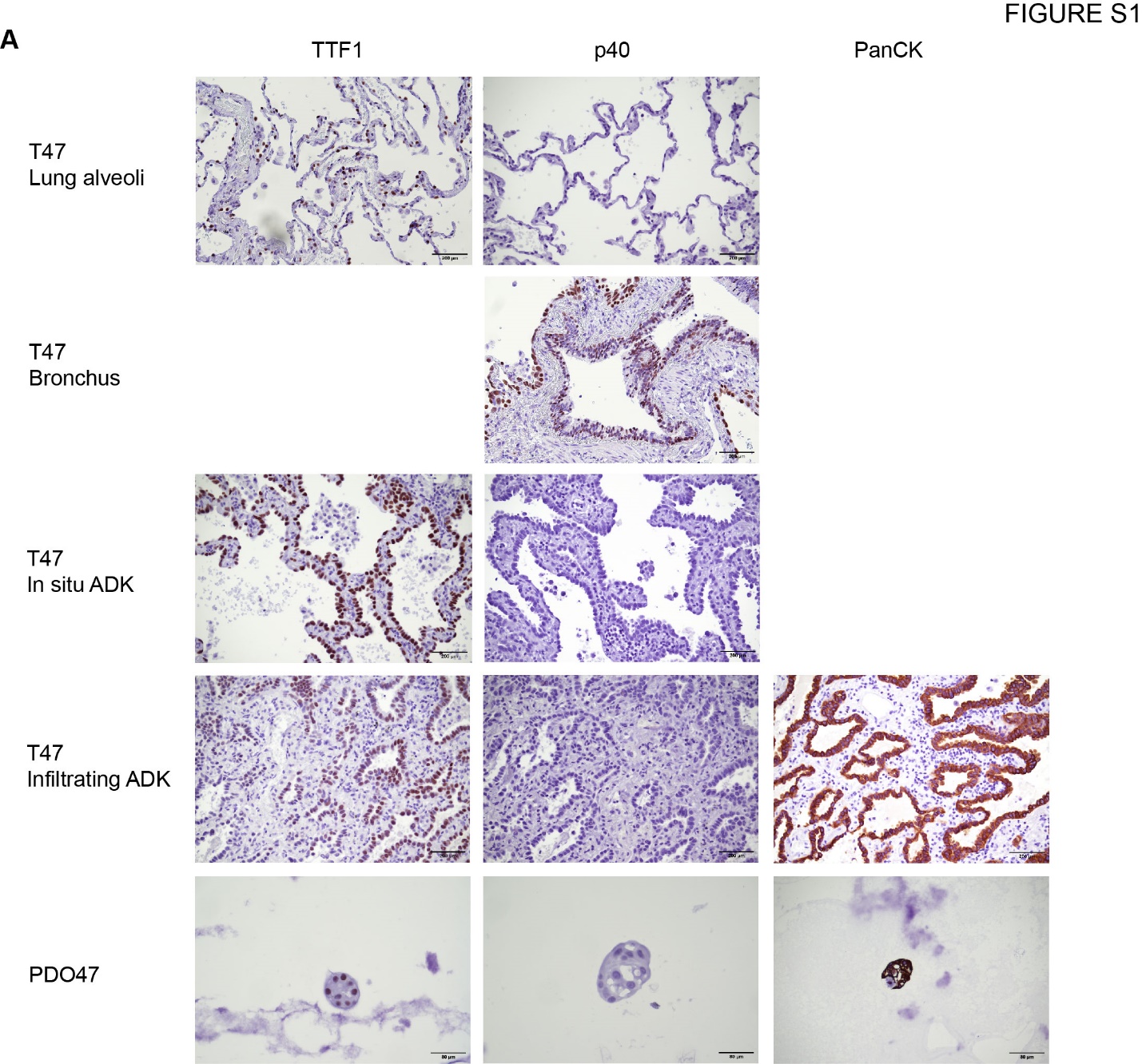
**

**Supplementary Figure 1.** **IHC staining controls**

Immunohistochemistry staining controls, performed on patient T47 and PDO47. TTF1 shows positivity in healthy lung alveoli and in lung adenocarcinoma (ADK), while p40 shows positivity in bronchial basal cells but negativity in adenocarcinoma. PDO47 showed good concordance with tumor tissue T47.

**
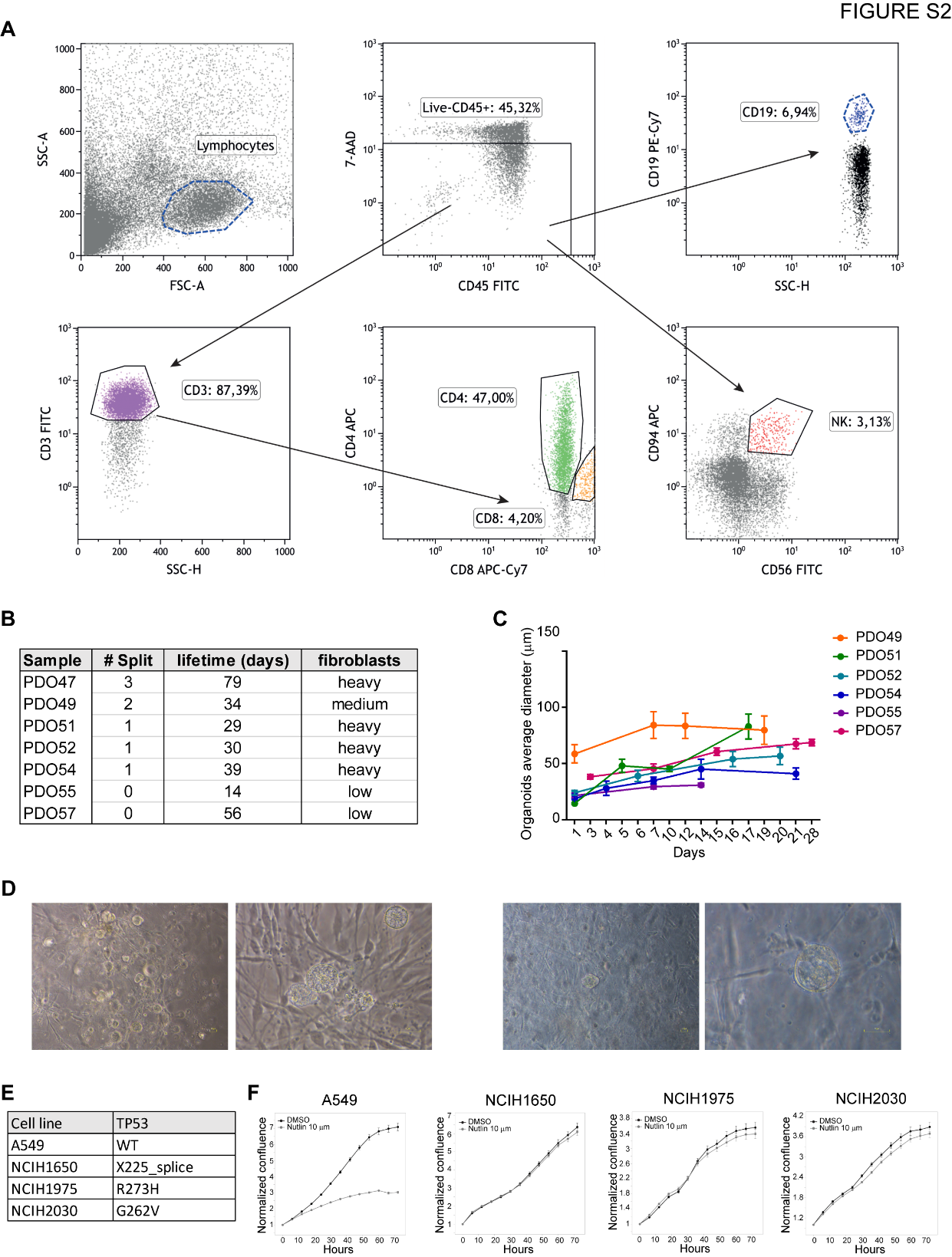
**

**Supplementary Figure 2.** **Additional controls**

A) Gating strategy for TILs analysis. B) Table indicating lifespan and fibroblast contamination of PDO cultured in Kim medium. C) Growth curves within the first split of PDO cultured in Kim medium. D) Pictures at different magnification (100x and 400x) of PDO51 and PDO52, grown in Kim medium and showing massive fibroblast contamination. E) TP53 mutational status of a panel of NSCLC cell lines. F) Nutlin-3a sensitivity in the panel of NSCLC cell lines. Confluence normalized on T0 is shown. Values are expressed as mean ± SEM (Standard error of the mean) (n=4; technical replicates).
