# Supplementary figures and images for: A Two-Step Protocol for Isolation and Maintenance of Lung Cancer Primary 3D Cultures
Source: Cancers (Basel). 2024 Dec 25;17(1):27. doi: 10.3390/cancers17010027 (PMC11718983; doi:10.3390/cancers17010027)

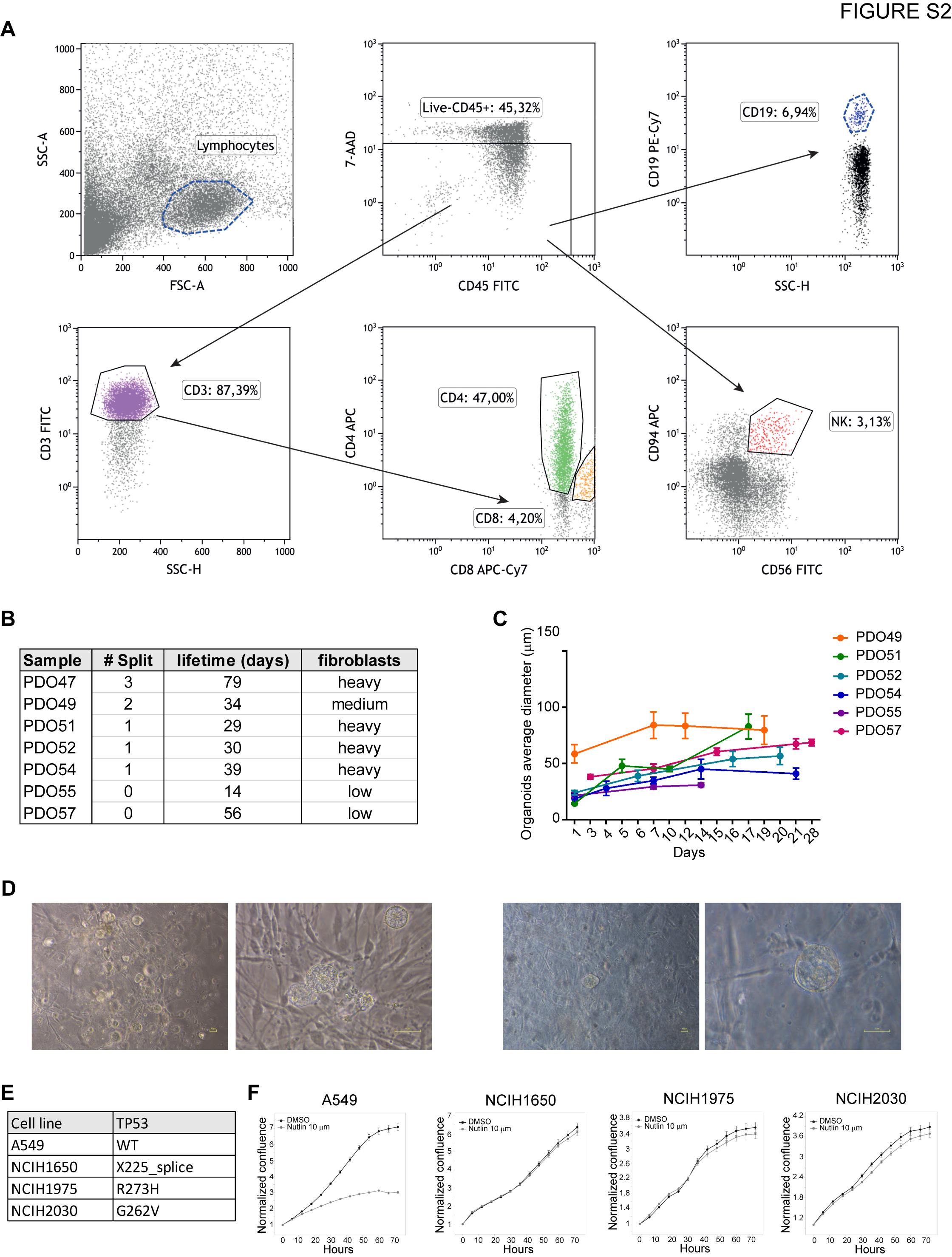

Supplement: Supplementary file 1 [file cancers-17-00027-s001.zip › Supplementary Figure 2.tif]

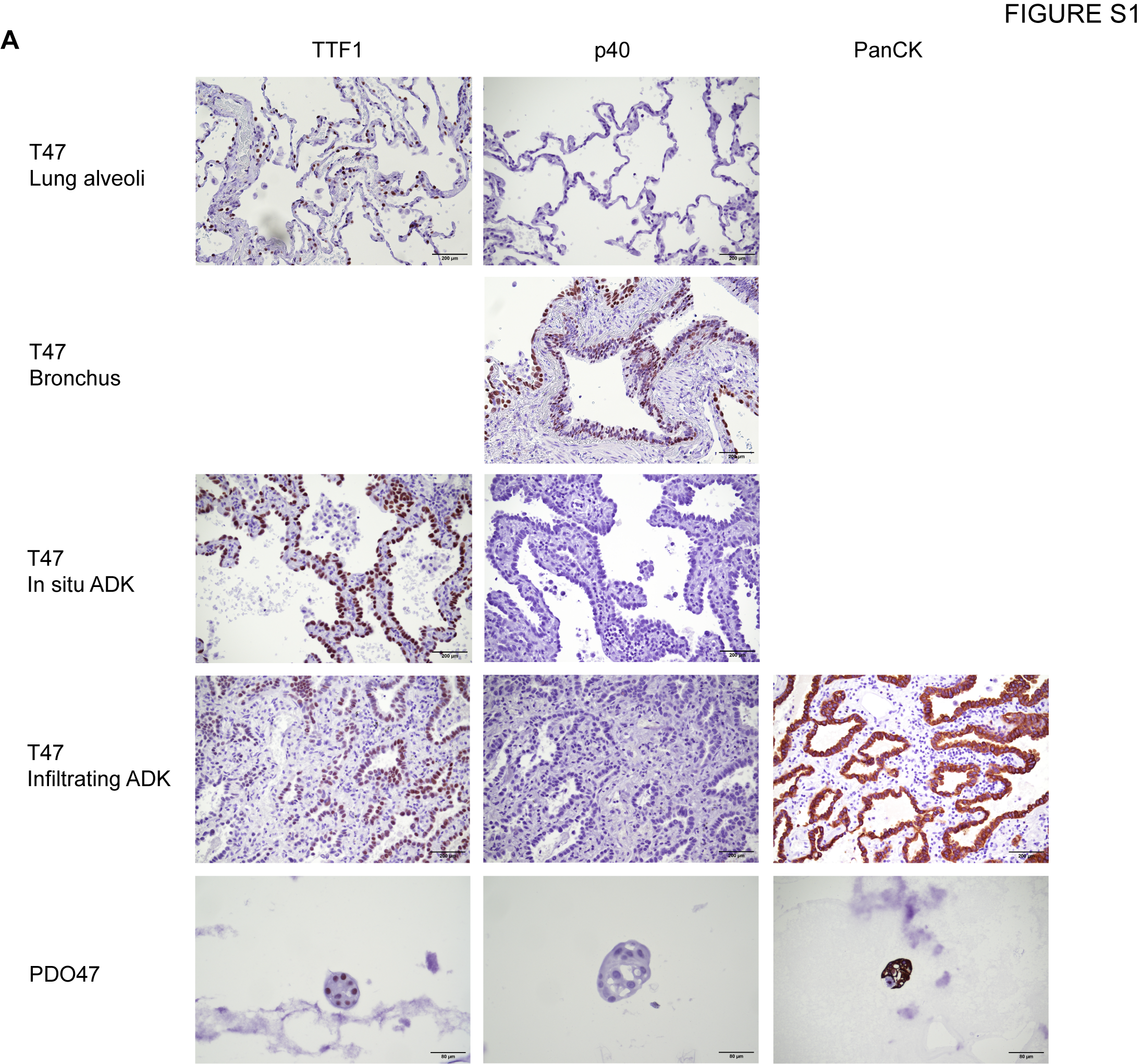

Supplement: Supplementary file 1 [file cancers-17-00027-s001.zip › Supplementary Figure 1.tif]
